# Supplementary material for: Engineering exosomes with iRGD for targeted RNAi therapy against pancreatic cancer mediated by long non-coding RNA PLBD1-AS1
Source: PLoS One. 2026 Apr 8;21(4):e0345697. doi: 10.1371/journal.pone.0345697 (PMC13061246; doi:10.1371/journal.pone.0345697)
Supplement: S1 Fig — (A) Survival analysis of ALDOA in the TCGA cohort. Quantitative analysis of Western blot for ALDOA and β-actin in MIA PaCa-2 and PANC-1 cell lines (n = 3). Extracellular acidification rate (ECAR) and oxygen consumption rate (OCR) in MIA PaCa-2 and PANC-1 cells. Quantitative analysis of AMPK pathway protein phosphorylation following PLBD1-AS1 knockdown (n = 3). Quantitative analysis of Western blot of phosphorylated AMPK pathway proteins in PANC-1 with single or dual knockdown of PLBD1-AS1 and ATP6V1B2 (n = 3). Statistical analyses were performed by Student’s t test (A and D) and one-way ANOVA (E). Bar graphs represent mean ± SEM. ns, not significant. *p < 0.05, **p < 0.01, ***p < 0.001. (PDF) [file pone.0345697.s001.pdf]

FIG S1

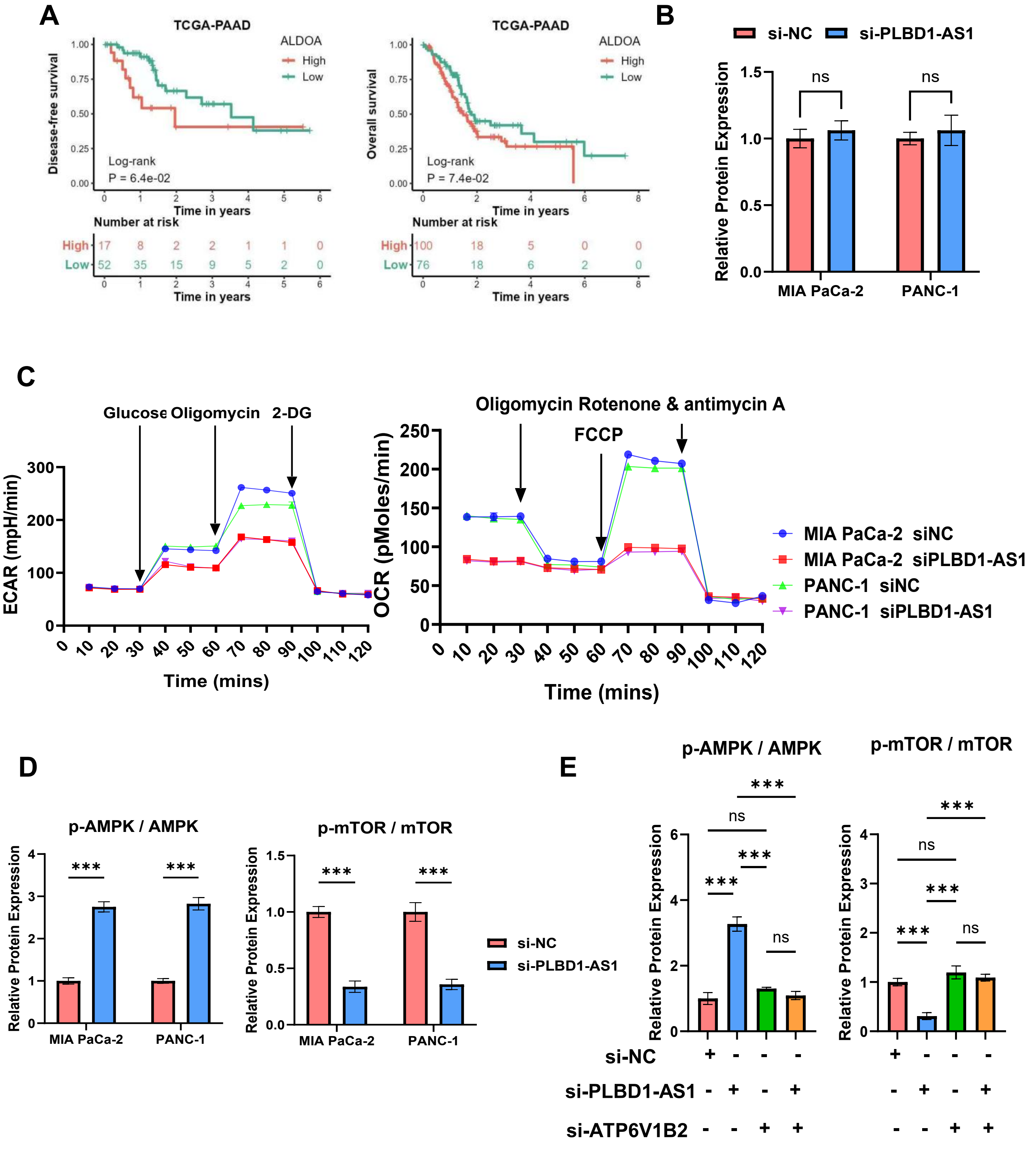
A
TCGA-PAAD
ALDOA
High
Low
Disease-free survival
Log-rank
P = 6.4e-02
Time in years
Number at risk
High 17 8 2 2 1 1 0
Low 52 35 15 9 5 2 0
TCGA-PAAD
Overall survival
Log-rank
P = 7.4e-02
High 100 18 5 0 0
Low 76 18 6 2 0
B
si-NC
si-PLBD1-AS1
Relative Protein Expression
ns
MIA PaCa-2
PANC-1
C
Glucose
Oligomycin
2-DG
ECAR (mpH/min)
Time (mins)
Oligomycin
Rotenone & antimycin A
FCCP
OCR (pMoles/min)
MIA PaCa-2 siNC
MIA PaCa-2 siPLBD1-AS1
PANC-1 siNC
PANC-1 siPLBD1-AS1
D
p-AMPK / AMPK
p-mTOR / mTOR
***
E
si-NC
si-PLBD1-AS1
si-ATP6V1B2
