# Supplementary figures and images for: Engineering exosomes with iRGD for targeted RNAi therapy against pancreatic cancer mediated by long non-coding RNA PLBD1-AS1
Source: PLoS One. 2026 Apr 8;21(4):e0345697. doi: 10.1371/journal.pone.0345697 (PMC13061246; doi:10.1371/journal.pone.0345697)

FIG S2

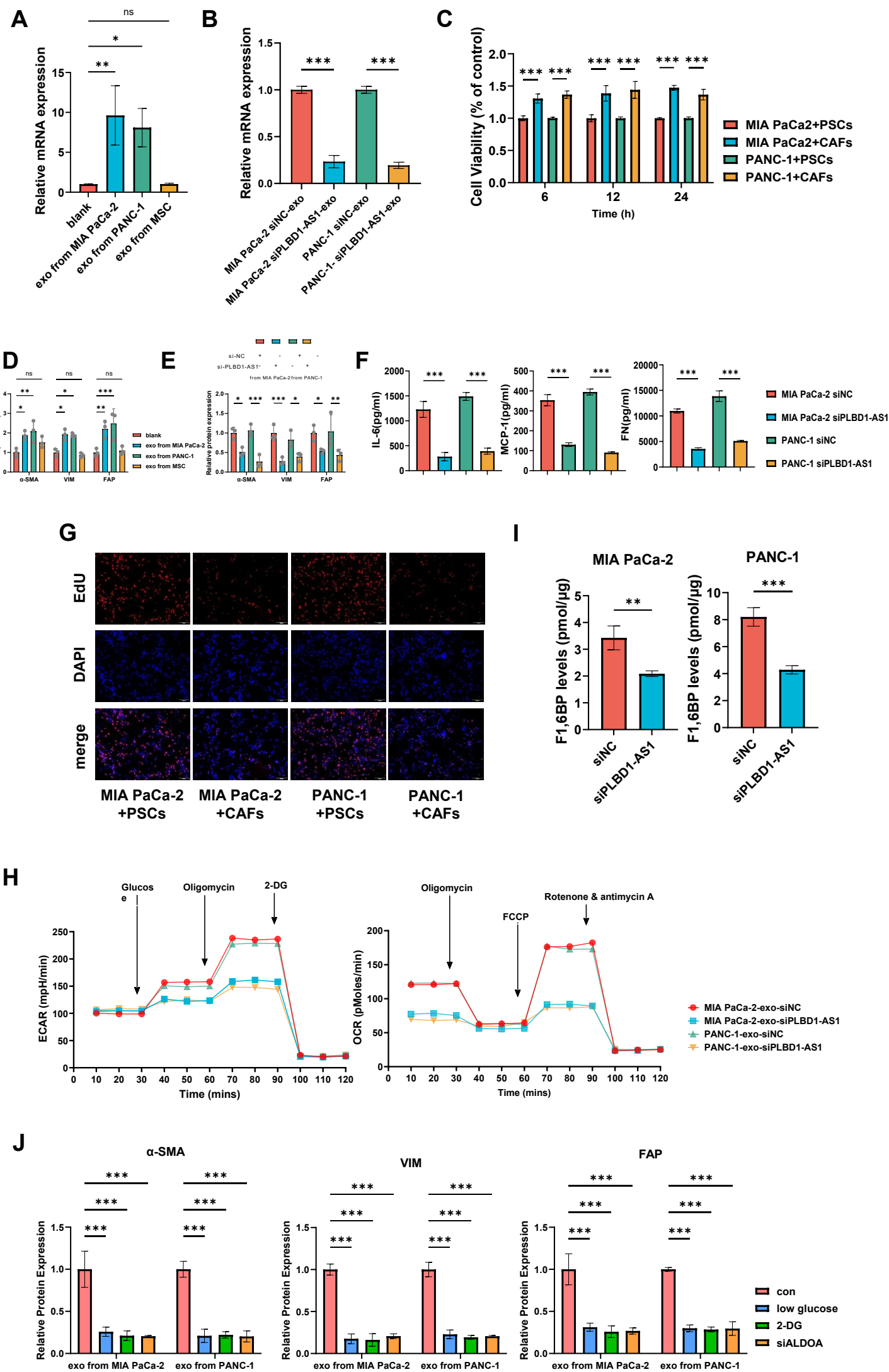

Supplement: S2 Fig — (A, B) Relative PLBD1-AS1 mRNA expression in PSCs after exosome treatment (n = 3).(C) Viability of MIA PaCa-2 and Panc1 cells co-cultured with PSCs or CAFs, assessed by CCK-8 assay (n = 3). (D, E) Quantitative analysis of α-SMA, VIM, FAP, and β-actin protein expression in PSCs following treatment with tumor cell-derived exosomes, as determined by Western blot (n = 3). (F) Secreted levels of IL-6, MCP-1, and FN in MIA PaCa-2 and Panc-1 cell supernatants, measured by ELISA (n = 3). (G) Representative images and quantitation of EdU assay showing proliferation of MIA PaCa-2 and PANC-1 cells co-cultured with PSCs 0or CAFs. (H) Extracellular acidification rate (ECAR) and oxygen consumption rate (OCR) of PSCs following treatment with MIA PaCa-2- or PANC-1-derived exosomes. (I) Fructose-1,6-bisphosphate (F1,6 BP) levels in PSCs after treatment with tumor cell-derived exosomes (n = 3). (J) Quantitative analysis of α-SMA, VIM, FAP, and GAPDH in PSCs incubated with exosomes under low glucose, 2-DG, or ALDOA knockdown conditions (n = 3). Statistical analyses were performed by Student’s t test (B, C, E, F, I and J) and one-way ANOVA (A and D). Bar graphs represent mean ± SEM. ns, not significant. *p < 0.05, **p < 0.01, ***p < 0.001. (PDF) [file pone.0345697.s002.pdf]

**FIG S3**

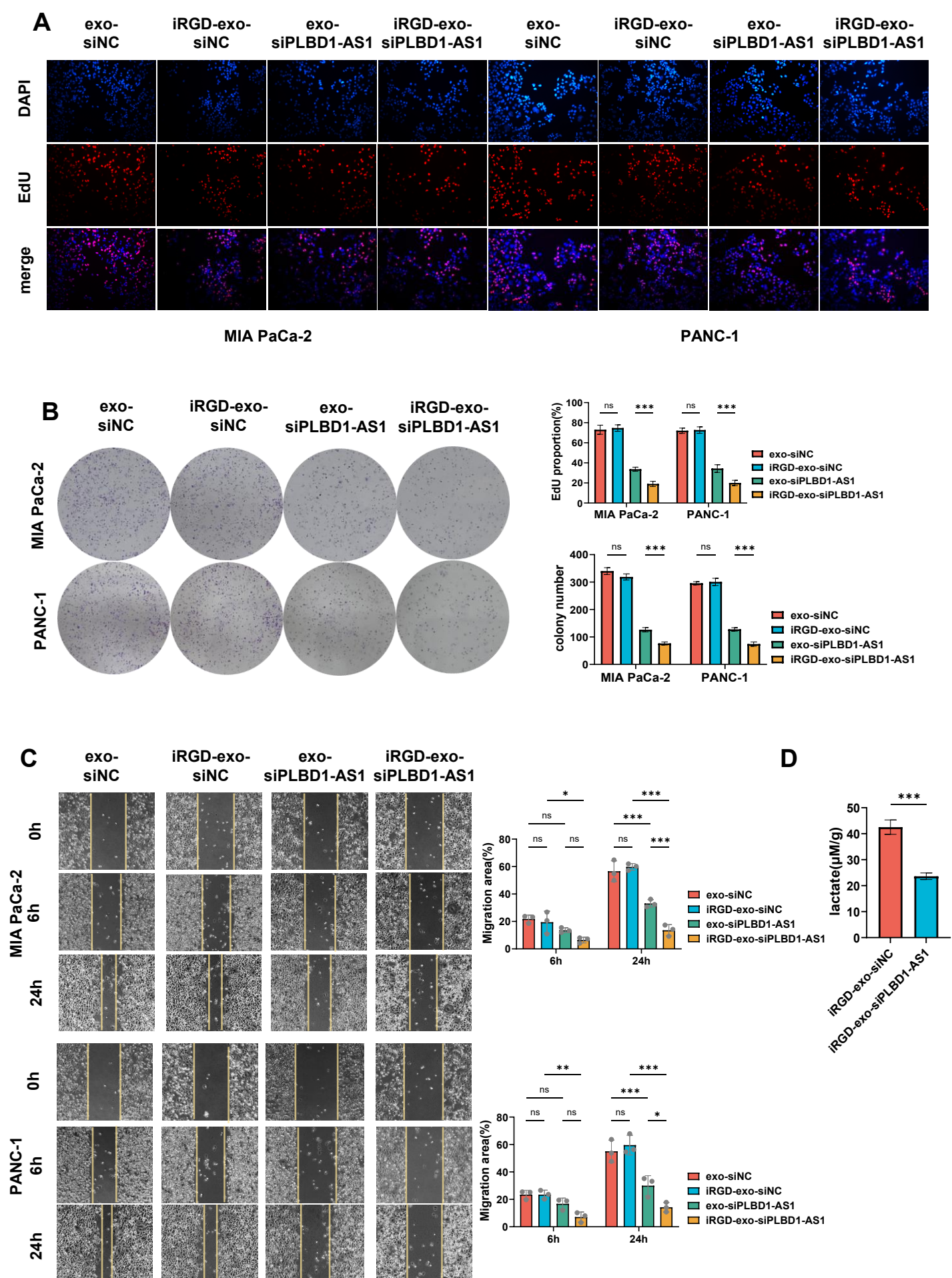

Supplement: S3 Fig — (A-C) Proliferation, clonogenicity, and migration of MIA PaCa-2 and PANC-1 cells treated with the indicated exosomes, as assessed by (A) EdU, (B) colony formation, and (C) wound healing assays (n = 3). (D) Lactate concentration in tumor tissues from nude mice treated with iRGD-exo-siNC or iRGD-exo-siPLBD1-AS1 (n = 3). Statistical analyses were performed by Student’s t test (B and D) and one-way ANOVA (C). Bar graphs represent mean ± SEM. ns, not significant. *p < 0.05, **p < 0.01, ***p < 0.001. (PDF) [file pone.0345697.s003.pdf]
