# Supplementary material for: Engineering exosomes with iRGD for targeted RNAi therapy against pancreatic cancer mediated by long non-coding RNA PLBD1-AS1
Source: PLoS One. 2026 Apr 8;21(4):e0345697. doi: 10.1371/journal.pone.0345697 (PMC13061246; doi:10.1371/journal.pone.0345697)
Supplement: Attachment — Submitted filename: Response to Reviewer 3.docx [file pone.0345697.s005.docx]

**Response to Reviewer 3 Comments**

We thank Reviewer 3 for their positive feedback and valuable suggestions to improve our manuscript. We have addressed all comments as detailed below, and all changes have been incorporated into the revised manuscript.

1. We have carefully checked the entire manuscript and corrected all instances of the typo "PLBD-as1" to the accurate nomenclature "PLBD1-AS1".
2. The exosomal RNA-seq dataset was sourced from the public repository GEO (accession number GSE175436). We have now explicitly stated this source and provided a description of the dataset in the 'Materials and Methods' section of the revised manuscript, clarifying that it contains exosomal lncRNA and mRNA expression profiles from 5 PDAC patients and 5 healthy donors. Regarding the more detailed patient screening strategy and specimen collection/processing methods, we note that this specific information was not provided by the original data submitters.
3. The values of "10,000 g" and "12,000 g" were indeed typographical errors. We have corrected them to **"100,000 g"** and **"120,000 g"** respectively in the revised manuscript.
4. We have now supplemented the relevant methods section with the requested key references that informed our experimental design for engineering targeted exosomes. Furthermore, we have provided a detailed, step-by-step description of the iRGD conjugation protocol in the revised 'Materials and Methods'.
5. We have carefully checked the entire Results section and corrected all erroneous figure citations to ensure they accurately correspond to the intended figures in the revised manuscript.
6. We have repeated the experiment for Figure 5E, now including both exo-siNC (non-targeting control exosomes) and iRGD-exo-siNC (iRGD-modified, non-targeting control exosomes) as essential control groups. The results, presented in the revised Figure 5E, clearly demonstrate the specific knockdown efficacy of siPLBD1-AS1 and the enhanced delivery benefit conferred by the iRGD modification compared to both control groups.
7. We agree that quantitative analysis strengthens the uptake data. Accordingly, we have performed flow cytometry to quantitatively assess the cellular uptake of DiL-labeled exosomes. These new quantitative data, which confirm the enhanced uptake of iRGD-exos, have been added as panel G in the revised Figure 5 and are described in the corresponding Results section.
8. We acknowledge that our current study, focused on elucidating the PLBD1-AS1/ALDOA/glycolysis axis, did not experimentally explore the functional relationship between PLBD1-AS1 and its sense gene PLBD1. We have now added a paragraph in the Discussion section to explicitly acknowledge this point, discuss the existing literature on PLBD1, and propose the investigation of its potential role and interaction with PLBD1-AS1 as an important direction for future research.

We believe these revisions have significantly improved the clarity, robustness, and completeness of our manuscript. We thank the reviewer again for their constructive input.
