# Supplementary material for: Engineering exosomes with iRGD for targeted RNAi therapy against pancreatic cancer mediated by long non-coding RNA PLBD1-AS1
Source: PLoS One. 2026 Apr 8;21(4):e0345697. doi: 10.1371/journal.pone.0345697 (PMC13061246; doi:10.1371/journal.pone.0345697)
Supplement: Attachment — Submitted filename: Response to Reviewers 2.docx [file pone.0345697.s006.docx]

Dear Reviewer,

Thank you for giving us the opportunity to submit a revised version of our manuscript. We sincerely appreciate the time and effort you have dedicated to reviewing our work and providing further insightful comments. We have carefully considered all the suggestions and have made corresponding revisions to the manuscript. Below, we provide a point-by-point response to each comment raised in the second round of review.

1.We performed multiple testing correction for our analyses, and this has been stated in the revised manuscript as the following: The p-values were adjusted for multiple testing using the Benjamini-Hochberg false discovery rate (FDR) method, and DEGs were defined with the following thresholds: adjusted p (FDR) < 0.05 and |log2Fold Change (FC)| > 1. PLBD1-AS1 was selected for further investigation because it emerged as the most significant prognostic factor in both the univariate Cox regression and Kaplan-Meier analyses within the TCGA dataset.

2.We appreciate the reviewer's valuable suggestions regarding the validation of the prognostic model. Indeed, developing and testing the model within the same TCGA cohort is a limitation. However, access to additional cohorts containing both lncRNA sequencing and detailed prognostic information for PDAC is extremely limited. Furthermore, the multivariate Cox analysis that included clinical variables did not yield supportive results for the independent prognostic value of our gene signature. Considering that the prognostic model itself does not affect the core conclusion of our study—which focuses on the functional role of PLBD1-AS1 in pancreatic cancer—we have decided to remove the original prognostic model section and the corresponding figures (previously Fig. S1A-D) from the manuscript.

3.We have incorporated the RIP-qPCR results into Figure 3B and provided a corresponding description in the text. The RIP-qPCR data further confirm the interaction between PLBD1-AS1 and ALDOA.

4.We appreciate the reviewer's constructive comment. We have performed additional experiments to evaluate the AMPK pathway. Given that the AMPK-mTOR axis is a central downstream pathway governing cellular energy sensing and growth, we analyzed the status of mTOR and p-mTOR. The results, included as new Figure 3H and 3I, show that alterations in ALDOA expression correspondingly affect mTOR/p-mTOR signaling. This evidence directly links our observed changes in AMPK activity to a key functional downstream pathway, thereby providing stronger support for the proposed ALDOA-vATPase-AMPK signaling cascade.

5.We thank the reviewer for the suggestion. To provide direct in vivo evidence for PLBD1-AS1-mediated activation of PSCs into CAFs, we have performed additional immunohistochemical staining for CAF marker, α-SMA, in our animal models. These data have been added to the revised manuscript.

6.We agree with the reviewer that the subcutaneous model has inherent limitations in replicating the complex tumor microenvironment of pancreatic cancer. We have expanded the Discussion section to more explicitly address this point and to provide stronger justification for our chosen exosome dosing regimen: While our subcutaneous xenograft model provided valuable initial insights into the in vivo efficacy of iRGD-exo-siPLBD1-AS1, we acknowledge that this model does not fully recapitulate the complex tumor microenvironment (TME) and metastatic propensity of human PDAC. The subcutaneous location lacks the organ-specific stroma, vascular network, and immune context of the pancreas. Future studies employing orthotopic pancreatic models or patient-derived xenografts (PDX) would be highly valuable. An orthotopic model would allow for the assessment of tumor growth and therapy response within the appropriate anatomical and microenvironmental context, while PDX models, which retain the stromal and genetic heterogeneity of human tumors, could provide even more clinically relevant data on the therapeutic potential and stromal remodeling capabilities of our engineered exosomes.

Besides, the exosome dosage used in this study (100 μg of iRGD-exo administered every two days for two weeks) was selected based on established protocols from recent preclinical studies. For example, comparable regimens include: 50 μg exosomes weekly in a hepatocellular carcinoma model (Zhang et al., J Hepatol. 2020)[1], 200 μg exosomes every two days in an angiogenesis study (Wang et al., Nat Commun. 2022)[2], and a dose of 5 mg/kg every three days in a lung cancer metastasis model (Li et al., J Control Release. 2021)[3]. Our regimen aligns with these reported safe and effective dosing schedules and showed no signs of acute toxicity in the current study."

7.We agree that systemic and immune safety assessments are crucial for establishing the therapeutic potential of engineered exosomes. In direct response to your suggestion, we have conducted additional experiments to evaluate both systemic and immune-related safety parameters. We monitored body weight throughout the treatment period. No significant weight loss was observed in the iRGD-exo-siPLBD1-AS1 treatment group compared to the control groups. Besides, We measured serum levels of the key pro-inflammatory cytokines IL-6 and TNF-α at the study endpoint. The results showed no significant elevation of these cytokines in the treatment group. These results have been added to Figure 5J and described in the corresponding text.

8.We have systematically checked and corrected the nomenclature for PLBD1-AS1 to ensure consistency.

9.Thank you for your careful review and for pointing out the need for language polishing. We have carefully proofread the manuscript and made thorough revisions to address minor grammatical and stylistic issues.

10.Thank you for the constructive suggestion. We agree that “oncogenic lncRNA” more precisely describes the functional evidence presented in our study. Accordingly, we have replaced “oncogene” with “oncogenic lncRNA” throughout the revised manuscript.

11.Thank you for raising this critical point. To address it, we have supplemented the “Materials and Methods” section with explicit statements on our practices for biological repeats, random allocation of samples/animals, and blinding during data acquisition and analysis. These details, along with the exact replicate numbers, are now also provided in each relevant figure legend.

12.Thank you for the valuable suggestion regarding the improvement of our Western blot figures. Clear molecular weight markers and quantitative information have been added to the revised manuscript.

We believe that the manuscript has been significantly improved thanks to your valuable suggestions. Please let us know if any further modifications or clarifications are needed. We look forward to hearing from you regarding the outcome of our revision.

Sincerely,

Xiangyu Kong

Department of Gastroenterology, Changhai Hospital, Naval Military Medical University, Shanghai, China.

xiangyukong185@hotmail.com

1.	Lou G, Chen L, Xia C, Wang W, Qi J, Li A, et al. MiR-199a-modified exosomes from adipose tissue-derived mesenchymal stem cells improve hepatocellular carcinoma chemosensitivity through mTOR pathway. J Exp Clin Cancer Res. 2020;39: 4. doi:10.1186/s13046-019-1512-5

2.	Zhang H, Mao Y, Nie Z, Li Q, Wang M, Cai C, et al. Iron Oxide Nanoparticles Engineered Macrophage-Derived Exosomes for Targeted Pathological Angiogenesis Therapy. ACS Nano. 2024;18: 7644–7655. doi:10.1021/acsnano.4c00699

3.	Jiang J, Lu Y, Chu J, Zhang X, Xu C, Liu S, et al. Anti-EGFR ScFv functionalized exosomes delivering LPCAT1 specific siRNAs for inhibition of lung cancer brain metastases. J Nanobiotechnology. 2024;22: 159. doi:10.1186/s12951-024-02414-7
